# Mood as a mediator of the link between child sexual abuse and psychosis

**DOI:** 10.1007/s00127-014-0966-1

**Published:** 2014-10-12

**Authors:** S. Marwaha, P. Bebbington

**Affiliations:** 1 Division of Mental Health and Wellbeing, Warwick Medical School, University of Warwick, Coventry, CV47AL UK; 2 Division of Psychiatry, University College London, 67-73 Riding House St., London, W1W7EJ UK

**Keywords:** Psychosis, Sexual abuse, Mediation, Affective disorder, Population survey

## Abstract

The significance of affective changes in psychosis is increasingly acknowledged, as is the role of early traumatic events. In a previous paper, using data from the English Adult Psychiatric Morbidity Survey 2007 (APMS2007), strong associations between child sexual abuse (CSA) and psychosis were demonstrated, with some evidence of mediation by affect. In the current paper, we subjected the same dataset to formal tests of mediation. For CSA involving sexual intercourse, 38.5 % of the link was mediated, 30.0 % by depression and 8.5 % by anxiety. For all forms of contact abuse, 38.2 % was mediated, 29.1 % by depression and 9.1 % by anxiety.

## Introduction

The separation of psychotic conditions from disorders regarded as having primary changes in mood is central to psychiatric classification systems. Recently, however, there has been a growing realisation of the significance of affective changes in psychosis. Anxiety and depression are both associated with paranoid ideation and auditory hallucinosis [1]. Conversely, people with anxiety and depressive disorders are more prone to psychotic-like experiences [2]. Finally, affective instability is strongly associated with psychosis, with the possibility that this may be responsible for fluctuations in psychotic symptoms [3].

The link between psychotic and affective disorders might arise because they share experiential antecedents. Indeed the affective response to stressful or traumatic events may drive the development of psychotic symptoms. Sexual abuse is a form of trauma that is strongly associated with a wide range of psychiatric disorders [4]. In an earlier paper, Bebbington et al. [5] demonstrated that, in its various forms, childhood sexual abuse is linked to psychosis. Controlling for contemporaneous levels of anxiety and depression (in the week prior to interview) reduced the association, but formal methods for quantifying the degree of putative mediation were not used. In the current paper, we subject the same dataset to a more rigorous procedure for establishing mediation.

## Method

General descriptions of the design and methods used in the 2007 Adult Psychiatric Morbidity Survey have been provided elsewhere [6], and the methods relating specifically to the analysis of sexual abuse are described in more detail by Bebbington et al. [5, 7]. The survey sought to establish a random sample of household residents aged 16 and above, representative of the adult population of England (5,689 of 7,461 participants provided sufficient data for these analyses). First phase interviewing was carried out by experienced non-clinical interviewers; while in a second phase, selected participants were interviewed clinically, in particular with the Schedules for Clinical Assessment in Neuropsychiatry (SCAN, version 2.1) [8].

Affect was assessed in relation to the preceding week during phase 1, using the Clinical Interview Schedule (revised) (CIS-R) [9]. This can be used to create overall scores for anxiety (range 0–8) and depressive symptoms (range 0–9). A category of probable psychosis included people diagnosed as such in the second phase, plus participants who did not attend the second phase but met at least two of four screening criteria based on the Psychosis Screening Questionnaire [10], antipsychotic medication use and medical contact with a given diagnosis of psychosis or schizophrenia. A detailed history of sexual abuse was obtained in phase 1, using confidential computer-assisted self-completion to encourage disclosure. In the current analysis, we use information about sexual abuse occurring before the age of 16 years: three levels of abuse were recorded, sexual talk (reported by 10.3 % of the sample), physical molestation (reported by 8.2 %), and non-consensual sexual intercourse (reported by 1.9 %). The commonest form, sexual talk, is more subjective than the first two, and is generally less strongly associated with psychiatric outcomes [4]. In the current analysis, we provide data separately for non-consensual sexual intercourse, and for “contact abuse”, a category derived by combining intercourse and unwanted sexual touching. Survey data were weighted to take account of survey design and non-response, to render the results representative of the English household population.

In the current paper, we used the ‘survey’ commands in STATA (Version 13 for MAC): these allow for the use of clustered data modified by probability weights, and provide robust estimates of variance. To test the role of affect as a mediator of the relationship linking CSA with psychosis, we applied the Karlson Holm Breen (-khb-) command in Stata. This method of mediation analysis decomposes the total effect of a variable into direct and indirect effects [11] and can be used in logit models. In these analyses, the confounding effects of concomitants on the decomposition were controlled for. These concomitants comprised sex, age, ethnicity, educational qualifications, equivalised household income, and whether the participant had been brought up by both biological parents until 16 years of age. Confidence limits were derived using the delta method of Sobell [12].

## Results

In Table 1, we show in detail mediation analyses incorporating depression and anxiety scores together. We also analysed the separate effects of anxiety and depression, reported in summary. Whether indicated by non-consensual sexual intercourse or by all forms of contact abuse, nearly 40 % of the association between child sexual abuse and psychosis was mediated by our measures of affect. In the analysis of contact abuse, the total and indirect effects are highly significant, but the direct effect is so only at trend level, although the odds ratio still exceeded 2. If this is not an issue of statistical power, it implies a predominant role for the mediation effect. In the analyses where depression and anxiety were both entered, depression was responsible for three quarters of the mediation. When anxiety was assessed as a mediator on its own, it was responsible for about a quarter of the link between sexual abuse and psychosis. This suggests that a sizeable amount of the mediation apparently driven by anxiety is best interpreted as being the consequence of the overlap between anxiety and depression. In this respect, the current results are in line with the simpler and less definitive analyses carried out by Bebbington et al. [5]. The results for mediation are remarkably consistent across the two types of sexual abuse that we assessed.

**Table 1:** Mood as a mediator of the link between child sexual abuse and psychosis

Effect	OR	Robust standard error	*z*	*p* > *z*	95 % CI
Depression and anxiety as mediators of the effect of childhood sexual intercourse on psychosis^a^					
Total	9.84	5.22	4.31	0.0001	3.48–27.85
Direct	4.08	2.21	2.59	0.009	1.41–11.79
Indirect	2.41	0.49	4.30	0.0001	1.61–3.61
Depression and anxiety as mediators of the effect of childhood contact abuse on psychosis^b^					
Total	3.42	1.49	2.82	0.005	1.46–8.02
Direct	2.14	0.94	1.73	0.083	0.91–5.04
Indirect	1.60	0.16	4.74	0.0001	1.32–1.94

Data were weighted, and controlled for sex, age, ethnicity, educational qualifications, equivalised household income, and whether the participant had been brought up by both biological parents until 16 years of age

^a^38.5 % of the link was mediated, 30.1 % by depression and 8.5 % by anxiety. If depression and anxiety are entered in separate analyses, depression mediates 37.4 % of the link, and anxiety 20.4 %

^b^38.2 % of the link was mediated, 29.1 % by depression and 9.1 % by anxiety. If depression and anxiety are entered in separate analyses, depression mediates 37.1 % of the link, and anxiety 24.4 %

## Discussion

Despite controlling for relevant sociodemographic variables, our analyses provide corroboration for the role of affect in mediating the link between child sexual abuse and psychosis, and suggest that depression may be of more significance in this context than anxiety. We must acknowledge limitations to our use of the data, and these are fully discussed by Bebbington et al. [5]. Computer-assisted self-completion interviews generally elicit franker responses than face-to-face questioning, and participants in APMS2007 were informed that the interviewer would have no access to their answers. We must also draw attention to a central assumption of our use of measures of current affective state in the mediation analysis. This is that these state measures are adequate indicators of a *propensity* to dysphoria. Only the latter could stand as a mediator.

Recently, other researchers have found evidence for the mediation by affect of links between childhood abuse and later psychotic symptoms in epidemiological samples [13–16]. Our findings support the current targeting of affective responses to stressful experience in cognitive-behavioural approaches to the treatment of psychosis.
